# Functional Validation of the Cytochrome P450 Family *PgCYP309* Gene in *Panax ginseng*

**DOI:** 10.3390/biom14060715

**Published:** 2024-06-17

**Authors:** Yang Jiang, Gaohui He, Ruiqi Li, Kangyu Wang, Yi Wang, Mingzhu Zhao, Meiping Zhang

**Affiliations:** 1 College of Life Science, Jilin Agricultural University, Changchun 130118, China; jiangyangyang1031@163.com (Y.J.); 15594540908@163.com (G.H.); lrq972152210@163.com (R.L.); kangyu.wang@jlau.edu.cn (K.W.); yi.wang@jlau.edu.cn (Y.W.); 2 Jilin Engineering Research Center Ginseng Genetic Resources Development and Utilization, Jilin Agricultural University, Changchun 130118, China

**Keywords:** *Panax ginseng*, ginsenoside, cytochrome P450, genetic transformation, functional validation

## Abstract

Ginseng (*Panax ginseng* C. A. Meyer) is an ancient and valuable Chinese herbal medicine, and ginsenoside, as the main active ingredient of ginseng, has received wide attention because of its various pharmacological active effects. Cytochrome P450 is the largest family of enzymes in plant metabolism and is involved in the biosynthesis of terpenoids, alkaloids, lipids, and other primary and secondary plant metabolites. It is significant to explore more *PgCYP450* genes with unknown functions and reveal their roles in ginsenoside synthesis. In this study, based on the five *PgCYP450* genes screened in the pre-laboratory, through the correlation analysis with the content of ginsenosides and the analysis of the interactions network of the key enzyme genes for ginsenoside synthesis, we screened out those highly correlated with ginsenosides, *PgCYP309*, as the target gene from among the five *PgCYP450* genes. Methyl jasmonate-induced treatment of ginseng adventitious roots showed that the *PgCYP309* gene responded to methyl jasmonate induction and was involved in the synthesis of ginsenosides. The *PgCYP309* gene was cloned and the overexpression vector pBI121-PgCYP309 and the interference vector pART27-PgCYP309 were constructed. Transformation of ginseng adventitious roots by the *Agrobacterium fermentum*-mediated method and successful induction of transgenic ginseng hairy roots were achieved. The transformation rate of ginseng hairy roots with overexpression of the *PgCYP309* gene was 22.7%, and the transformation rate of ginseng hairy roots with interference of the *PgCYP309* gene was 40%. Analysis of ginseng saponin content and relative gene expression levels in positive ginseng hairy root asexual lines revealed a significant increase in PPD, PPT, and PPT-type monomeric saponins Re and Rg2. The relative expression levels of *PgCYP309* and *PgCYP716A53v2* genes were also significantly increased. *PgCYP309* gene promotes the synthesis of ginsenosides, and it was preliminarily verified that *PgCYP309* gene can promote the synthesis of dammarane-type ginsenosides.

## 1. Introduction

Ginseng is part of a long history of medicinal plants belonging to the *Araliaceae* family, whose main compounds include saponins, polysaccharides, polykynes, peptides, vitamins, organic acids, and trace elements among others [1]. Ginsenosides, the main pharmacologically active constituents in ginseng, are an important class of secondary metabolites formed by linking triterpene glycosides to sugar groups [2], and are categorized into an oleanocarpane-type and dammarane-type [3]. There is only one oleanocarpane-type, Ro, while the dammarane-type is subdivided into protopanaxadiol-type (PPD-Type) and protopanaxatriol-type (PPT-Type) depending on the glycoside [4]. Protopanaxadiol type saponins include Rb1, Rb2, Rb3, Rc, Rd, Rg3, and Rh2, etc.; and protopanaxatriol type saponins include Re, Rf, Rg1, Rg2, and Rh1, etc. [5]. Pharmacological studies have shown that ginsenosides have anti-inflammatory [6] and anticancer properties [7], and result in cardiovascular protection [8], improvement of fatigue and weakness [9], and enhancement of immunity [10], and now it has been widely used in agricultural products, food, health products and cosmetics [11]. However, ginseng grows slowly and requires high climatic and geographic conditions, and the traditional way of planting ginseng to produce saponins can no longer satisfy the growing market demand. Therefore, it is a major issue for the development of the ginseng industry to reveal the biosynthesis mechanism of ginsenosides and to innovate the production method of ginsenosides.

Ginsenosides are mainly synthesized through a combination of the mevalonate (MVA) pathway in the cytoplasm [12] and the methylerythritol phosphate (MEP) pathway in the plastid [13], which involves more than 20 consecutive steps of enzymatic reactions [14]. Enzymes in the middle of these reactions play a crucial role, but only a few key enzyme genes have been identified and cloned. Therefore, more in-depth studies around these key enzyme genes are important for refining and enriching the ginsenoside biosynthesis pathway.

Cytochrome P450 (CYP450) is an oxidase that plays a key role in the synthesis of important metabolites in living organisms [15] and is also the largest superfamily of supergenes in nature [16], which comprises more than 13,000 genes from over 400 gene families [17]. This family is widely distributed in plants, animals, and microorganisms and participates in a variety of biochemical pathways to produce primary and secondary metabolites [16], such as phenylpropanoids, alkaloids, terpenoids, and phytohormones [18,19,20]. In the triterpene saponin biosynthetic pathway, this family catalyzes structural modifications of the triterpene skeleton such as hydroxylation, carboxylation, aldolylation, ketonylation, and dehydrodehydration to form intermediates of triterpene saponins [21,22,23]. However, *P450* genes intervene in terpenoid biosynthesis, decorating the terpene hydrocarbon skeleton with mostly hydroxyl groups, but there are few studies on the involvement of the CYP450 family in ginsenoside biosynthesis.

Inducers can significantly increase the accumulation of secondary metabolites in plants in a short period of time, so they are often used to research the biosynthetic pathways of secondary metabolites in plants. Studies have shown that exogenous treatment with jasmonic acid derivatives (e.g., methyl jasmonate, MeJA) can affect the accumulation of triterpenoid saponins in plants. In *Panax ginseng*, *Centella asiatica*, *Panax notoginseng*, *Panacis Quinquefolii Radix* and *Chenopodium quinoa*, MeJA treatment was able to modulate the expression activities of key enzyme genes in plants in vivo and in isolated tissues, such as *FPS*, *SS*, *CYP450s*, and *UGTs*, in plant in vivo and in isolated tissues, which in turn affected the accumulation of saponins [24,25,26,27,28,29,30,31,32,33]. MeJA-induced treatment is the most common and effective way to study the function of key enzyme genes for ginsenoside synthesis and the mechanism of ginsenoside synthesis.

The laboratory has already mined the *CYP450* gene family in Jilin ginseng by means of bioinformatics and performed systematic analyses of evolution, structure, function, expression, and interactions, as well as obtaining five candidate genes related to the biosynthesis of ginsenosides [34]. In this study, based on the correlation analysis with saponin content and key enzyme interactions, we further screened and obtained a gene, *PgCYP309*, which is most closely related to saponin synthesis. We investigated the expression trend under the regulation of methyl jasmonate, performed gene overexpression and RNA interference, and transformed the adventitious roots of ginseng by an *Agrobacterium*-mediated method to preliminarily explore its role in the synthesis of ginsenosides. These investigations were carried out in order to provide genetic resources for the application of this gene family in ginseng, to lay the foundation for elucidating the molecular mechanism of ginsenoside biosynthesis and to innovate ginseng genetic resources.

## 2. Materials and Methods

### 2.1. Experimental Data and Materials

This study was based on the Jilin ginseng transcriptome database constructed by Jilin Engineering Research Center Ginseng Genetic Resources Development and Utilization [35]. The *PgCYP450* gene family was obtained from a pre-laboratory screen [34]. The ginseng receptor materials for MeJA-induced regulation and genetic transformation were induced cultured ginseng adventitious roots propagated on B5 solid medium in our laboratory. The vectors and organisms used in the experiment were stored in the laboratory.

### 2.2. Identification and Analysis of Candidate Genes

The expression of five *PgCYP450* genes was extracted from the database using Perl software. The expression of five *PgCYP450* genes in 42 farm cultivars and the expression of nine individual saponins and total saponins in 42 farm cultivars were analyzed by Pearson’s correlation coefficients using SPSS software and the correlation coefficients were statistically determined at *p* ≤ 0.05 and *p* ≤ 0.01 conditions. Set *p* value ≤ 0.05 for significant correlation and *p* value ≤ 0.01 for highly significant correlation. The expression data of five *PgCYP450* genes and ginsenoside synthesis key enzyme genes from 42 farm cultivars were collated, and the Spearman correlation coefficients between different genes were calculated by R. The interactions network was mapped by BioLayout Express3D. The final candidate genes were identified by synthesizing the results of the correlation analysis with saponins and the key enzyme interaction network analysis.

### 2.3. Bioinformatics Analysis of Candidate Genes

In order to lay the foundation for the subsequent in-depth study of the candidate gene, the protein properties, secondary structure and tertiary structure of the candidate genes were analyzed and predicted. The protein sequences of the candidate genes were uploaded into the ExPASy website for protein physicochemical property analysis; using the SOPMA website, the secondary structure of the proteins was predicted; the tertiary structure of the proteins was predicted in the SWISS-MODEL website; and the phosphorylation sites of the proteins were predicted in the NetPhos website.

### 2.4. Determination of Adventitious Roots and Saponin Content and Gene Expression in Ginseng under MeJA Treatment

Ginseng adventitious roots of 1.0 g grown for 25 days were inoculated in 250 mL of B5 liquid medium. They were incubated in a constant temperature shaker at 22 °C at 110 rpm for 25 days. After 25 days, methyl jasmonate was added to the shake flask at a final concentration of 200 μmol/L. Eleven treatment groups with different induction times (6 h, 12 h, 24 h, 36 h, 48 h, 60 h, 72 h, 84 h, 96 h, 108 h, and 120 h) and a control group without induction treatment were set up. Three biological replicates were set up for each group. At the end of the treatment, 1.0 g of adventitious roots was snap-frozen with liquid nitrogen and stored at −80 °C for reserve. The remaining adventitious roots were dried and used for saponin extraction.

RNA in treated ginseng adventitious roots was extracted using the Trizol method and reverse transcribed into cDNA. Reactions were performed using the fluorescence quantification kit to analyze the expression of the *PgCYP309* gene and the six key enzyme genes that have been validated in the laboratory (*PgDDS*, *PgCYP716A47*, *PgCYP716A53v2*, *PgCYP716A52v2*, *PgUGT1*, and *PgUGT100*). Three biological replicates were designed, and the final gene relative expression was calculated using the 2^−ΔΔCt^ formula. The saponins of the treated ginseng adventitious roots were extracted, and the content of total saponins was determined by the colorimetric method of vanillin-glacial acetic acid [36]. The methanol was used as a blank control and freshly configured vanillin-glacial acetic acid solution was added to the samples and the absorbance was finally measured at 544 nm. The content of ginseng monomeric saponins was determined by high performance liquid chromatography (HPLC). The ginsenoside content of the samples were calculated by applying the following formula: ginsenoside content (mg/g) = peak area of the sample/peak area of the standard × concentration of the standard × volume of the sample solution/dry weight of the sample.

### 2.5. Cloning of PgCYP309 Gene

The open reading frame (ORF) of the *PgCYP309* gene was found by NCBI, primers were designed using Primer 6.0 and then sent to the company for synthesis. Ginseng adventitious root RNA was extracted and reverse transcribed into cDNA using the SPARKscript II RT Plus kit (with gDNA Eraser) (Shandong Sparkjade Biotechnology Co., Ltd., Jinan, China). The cDNA was used as a template for the gene cloning, and the PCR products that matched the size were recovered with a gel recovery kit. The recovered fragments were ligated with T vector, transformed into *E. coli* sensory state, and the monoclonal colonies were picked for bacteriophage PCR verification. The successfully validated bacterial fluids were sent to the company for sequencing to ensure the accuracy of the gene sequences.

### 2.6. Construction of PgCYP309 Gene Overexpression Vector and Interference Vector

The cloning vector and the overexpression vector pBI121 were double digested using *BamH* Ⅰ and *Xba* Ⅰ, followed then by the ligation of the target gene fragments with the digested vector fragments. The transformed *E. coli* sensory state picked single clone colonies for bacterial liquid PCR verification and double enzyme digestion verification, and the selected positive strains sent to the company for sequencing. Interference fragment primers were designed, and PCR amplification was performed to obtain the positive and negative fragments of the *PgCYP309* gene. The pHANNIBAL-PgCYP309 recombinant plasmid was obtained by ligating the positive-sense and antisense fragments into the pHANNIBAL vector. The recombinant plasmid and pART27 vector were digested using *Not* Ⅰ enzyme and then ligated. After transforming the *E. coli* sensory state, single colonies were picked and verified by double enzyme digestion. The recombinant plasmids of the successfully ligated overexpression vectors and interference vectors were transformed into *Agrobacterium* and verified by bacteriophage PCR. It was determined to be consistent with the size of the target bands, and the bacterial fluid was preserved for subsequent genetic transformation experiments.

### 2.7. Genetic Transformation of Ginseng Adventitious Roots

Ginseng adventitious root materials with good growth were cut into segments, placed in MS medium plates, and light-cultured at 23 °C for 2 days to complete the pre-culture of adventitious root materials. The 200–400 μL of engineering bacteria with high growth activity was aspirated and added to 50 mL of liquid LB medium, and placed in a shaker at 28 °C, 170 rpm for overnight shaking culture. When the OD600 of the bacterial solution was 0.4–0.5, it was centrifuged at 6000 rpm for 5 min. The supernatant was discarded, and the precipitate was resuspended by adding 1/2 MS medium containing AS and brought to an OD_600_ value of 0.4–0.5. The bacterial solution was activated by placing it in a shaker at 28 °C and 50 rpm. Pre-cultured adventitious root material was cut into 0.4–0.5 mm segments and transferred to the activated bacterial solution for 15 min of infestation. After completion of the infestation, small segments were removed and blotted dry, transferred to 1/2 MS solid medium containing AS, and incubated for 2 days at 23 °C under dark conditions. After two days, the material was transferred to 1/2 MS medium containing cephalexin and incubated at 23 °C in dark conditions. The growth status was regularly observed and recorded until the growth of hairy roots.

### 2.8. Positive Material Detection and its Gene Expression Change and Saponin Content Change Analysis

The genomic DNA of ginseng hairy root was extracted by TPS method [37]. The samples were added to a 1.5 mL centrifuge tube with 200 μL of TPS extract, added and mashed, and placed in a 75 °C metal bath for 20 min. It was centrifuged at 12,000 rpm for 10 min at room temperature. The supernatant was aspirated into a new centrifuge tube and after adding an equal volume of isopropanol, it was left at room temperature for 10 min and centrifuged at 5000 rpm for 10 min. The supernatant was discarded and 200 μL of 70% ethanol was added to it centrifuged at 5000 rpm for 5 min. The supernatant was discarded, the precipitate was dried, and the DNA was solubilized by adding ddH_2_O. Then a three-stage PCR (target gene and fragment of vector upstream of target gene; target gene fragment; target gene and fragment of vector downstream of target gene) was performed. Positive material was extracted for RNA and reverse transcribed to cDNA. Specific primers were designed for fluorescence quantification and reactions were carried out using a fluorescence quantitative PCR kit. The final data results were obtained, and the data were calculated as 2^−ΔΔCt^ and plotted. Ginsenosides were extracted using Soxhlet extraction, and the saponin content was determined using high performance liquid chromatography (HPLC). The conditions used were as follows: the chromatographic column was a Water C18 column; the mobile phase consisted of acetonitrile and water; the sample volume was 20 μL each time, and the flow rate of the mobile phase was 1 mL per minute; the temperature of the column was at 35 °C; and its detection wavelength was set at 203 nm.

## 3. Results

### 3.1. Correlation Analysis between PgCYP450 Gene and Ginseng Saponin Content

**Figure 1:**
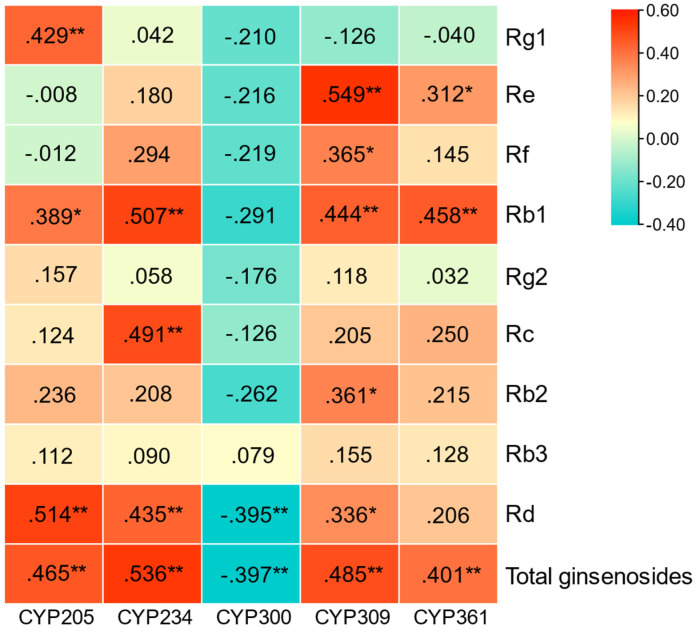
**Correlation analysis between *PgCYP450* gene and ginsenoside content.** “*” correlation indicates significance at the 0.05 level. “**” correlation indicates significance at the 0.01 level.

On the basis of previous laboratory studies [34], we obtained five *PgCYP450* family genes. The expression of five *PgCYP450* genes in 42 farm cultivars, nine individual saponins and total saponins in 42 farm cultivars were extracted and analyzed by Pearson’s correlation coefficient; the results are shown in Figure 1. All five genes were found to be significantly associated with at least one single saponin and highly significantly associated with total glycosides.

### 3.2. Interaction Analysis of PgCYP450 Gene with Key Enzyme Genes

**Figure 2:**
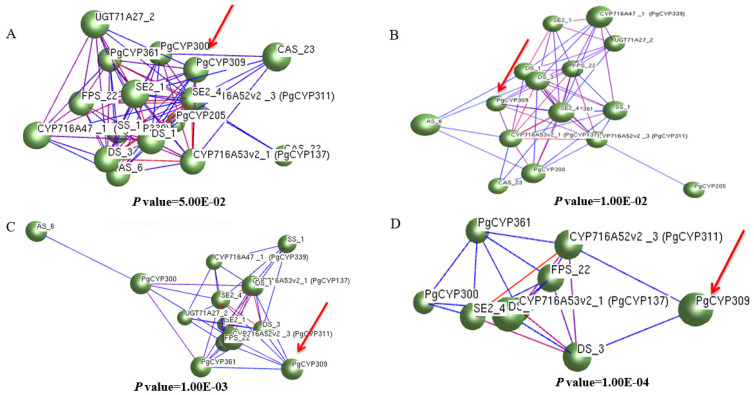
**Interaction between *PgCYP450* gene and key enzyme genes.** (**A**) The network was constructed at *p* ≤ 5.00 × 10^−2^. (**B**) The network was constructed at *p* ≤ 1.00 × 10^−2^. (**C**) The network was constructed at *p* ≤ 1.00 × 10^−3^. (**D**) The network was constructed at *p* ≤ 1.00 × 10^−4^.

Construction of co-expression interaction network using the expression of five *PgCYP450* genes in 42 different farm cultivars with 16 key enzyme genes. The results are shown in Figure 2. When *p* value ≤ 5.00 × 10^−2^ and *p* value ≤ 1.00 × 10^−2^, there were four *PgCYP450* genes clustered in the same cluster with the key enzyme genes as *PgCYP205*, *PgCYP300*, *PgCYP309*, and *PgCYP361*; when *p* value ≤ 1.00 × 10^−3^ and *p* value ≤ 1.00 × 10^−4^, three genes were clustered in the same cluster with the key enzyme genes as *PgCYP300*, *PgCYP309* and *PgCYP361*.

Meanwhile, combined with the results of correlation analysis with saponins, *PgCYP309* gene was highly significantly correlated with Re, Rb1 and total saponin content, and significantly correlated with Rf, Rb2 and Rd content, indicating that the *PgCYP309* gene is very likely to play an important role in the saponin synthesis pathway just as the key enzyme genes do, thus it was selected to carry out the next functional validation.

### 3.3. Bioinformatics Analysis of the PgCYP309 Gene

**Figure 3:**
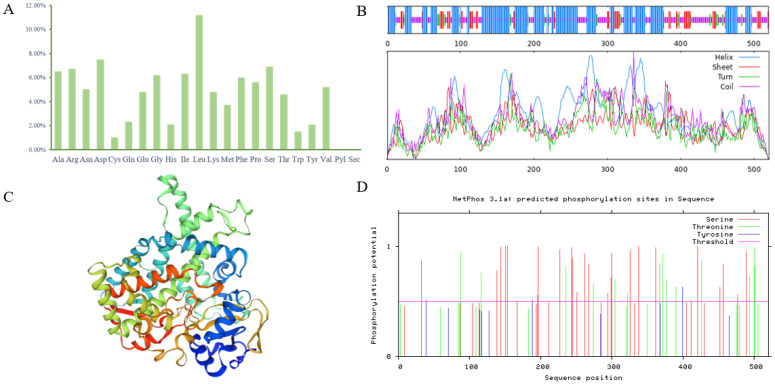
**Characterization analysis of the *PgCYP309* gene.** (**A**) Amino acid distribution of PgCYP309 protein. (**B**) Prediction of the secondary structure of PgCYP309 protein. (**C**) Tertiary structure prediction of PgCYP309 protein. (**D**) Prediction of PgCYP309 protein phosphorylation sites. Helix: alpha helix (blue); sheet: extended strand (red); turn: beta turn (green); coil: random coil (purple).

The *PgCYP309* gene was analyzed, and the *PgCYP309* gene contains one cytochrome p450 structural domain located between amino acids 244-1690 and belongs to the p450 superfamily. Its full-length cDNA sequence was 2121 bp, with an ORF length of 1563 bp, encoding 520 amino acids, of which Leu was the most abundant at 11.2%, and Pyl and Sec were the least at 0.0% (Figure 3A); the predicted molecular weight was 58.87 kDa; the relative molecular weight was 58,867.71; the isoelectric point was 6.29, acidic; the negatively charged residues included Asp and Glu with a total number of 64, and the positively charged residues included Arg and Lys with a total number of 60; the instability index of the gene was 38.65, a stable protein; the lipid index was 89.85; and the hydrophilicity mean value was -0.182, which is a hydrophobic protein. The results of the secondary structure prediction of the PgCYP309-encoded protein, includes α-helices (46.54%), β-turns (6.15%), extended strands (12.69%), and random coils (34.62%) (Figure 3B). Tertiary structure prediction results are shown in Figure 3C. As shown in Figure 3D, the number of amino acid residues presumed to bind to the ATP phosphate group are 25 for serine (Ser), 3 for tyrosine (Tyr), and 14 for threonine (Thr).

### 3.4. Analysis of Saponin Content and Gene Expression after MeJA-induced Treatment of Ginseng Adventitious Roots

**Figure 4:**
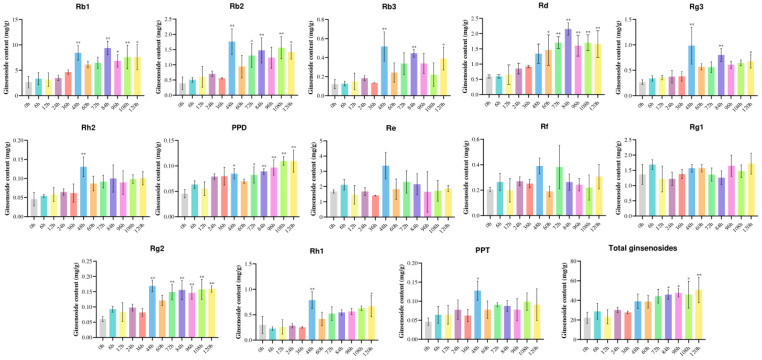
**Effect of MeJA induction on ginsenoside content in ginseng adventitious roots.** The X-axis indicates the processing time. The Y-axis indicates the ginsenoside content (mg/g). “*” indicates significant difference at *p* ≤ 0.05. “**” indicates significant difference at *p* ≤ 0.01.

After treating ginseng adventitious roots with MeJA, the saponins in ginseng adventitious roots were extracted, and the contents of 12 monomeric saponins, including Rb1, Rb2, Rb3, Rc, Rd, Re, Rf, Rg1, Rg2, Rg3, Rh1, Rh2, as well as two glycosides, PPD and PPT, were determined, and the total saponins were determined by the colorimetric method of vanillin-glacial acetic acid (Table S1). The calculated saponin content results are shown in Figure 4, where control (0 h) is the average saponin content of samples without MeJA-induced treatment. Except for Rc, which was not detected, most of the monomeric saponins, as well as the two glycosides, increased with increasing MeJA induction time. Only three monomeric saponins, Re, Rf, and Rg1, which are all PPT-type saponins, did not change significantly. The content of PPD-type saponins increased significantly after MeJA-induced treatment, while PPT-type saponins did not change significantly.

**Figure 5:**
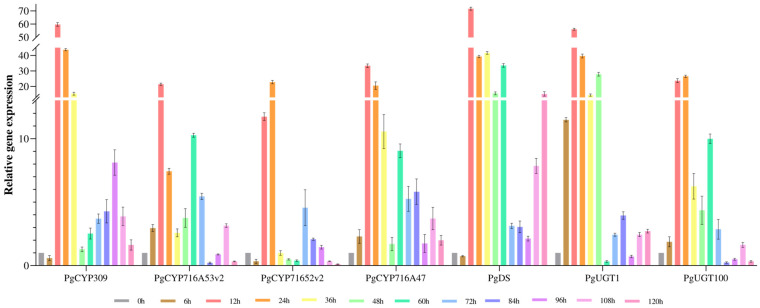
Expression levels of *PgCYP309* gene and six key enzyme genes of ginsenoside synthesis in response to MeJA treatment. The X-axis indicates the processing time. The Y-axis indicates the relative expression levels of genes.

The expression levels of the PgCYP309 gene with six key enzyme genes (*PgDDS*, *PgCYP716A47*, *PgCYP716A53v2*, *PgCYP716A52v2*, *PgUGT1*, and *PgUGT100*) were computationally analyzed using qRT-PCR (Figure 5). The results showed that the relative expression of *PgCYP309* gene and the six key enzyme genes were up-regulated, and all of them were most obvious at the induction treatment time of 12–24 h, indicating that the *PgCYP309* gene and the six key enzyme genes were responsive to MeJA induction. Combined with the results of 2.1 saponin content analysis, the *PgCYP309* gene may be involved in the synthesis of ginsenosides together with six key enzyme genes. This result simultaneously demonstrates the accuracy of candidate gene selection and lays the foundation for subsequent functional validation of the *PgCYP309* gene.

### 3.5. Cloning of PgCYP309 Gene and Construction of Vector

Ginseng RNA was extracted from the mixed ginseng samples and selected for reverse transcription with high purity. The *PgCYP309* gene was amplified using specific primers with cDNA as the template. Electrophoretic detection showed that the size of the bands was consistent with the size of the target gene. The gene was recovered, ligated and transformed with the cloning vector, and after the bacterial liquid PCR was verified, the positive strain was selected and sent to the company for sequencing. The sequencing results were consistent with the target sequence, and the *PgCYP309* gene was successfully cloned. The cloning vector ligated with the *PgCYP309* gene was double digested with the overexpression vector, recycled, ligated and transformed. The positive strain was selected and sent to the company for sequencing, the overexpression vector was constructed successfully, and the recombinant vector was named pBI121-PgCYP309. Positive and antisense fragments of gene disruption were amplified using specific primers and inserted into intermediate vectors. The interference fragment was cut down and ligated into pART27 vector, and after successful transformation, bacterial PCR was performed to verify that the interference vector was constructed successfully, and the recombinant vector was named pART27-PgCYP309. The overexpressed and interfering recombinant plasmids were transformed into *Agrobacterium*, and the bacterial fluids were preserved for subsequent genetic transformation experiments.

### 3.6. Genetic Transformation of Ginseng Explants and Detection of Positive Material

**Figure 6:**
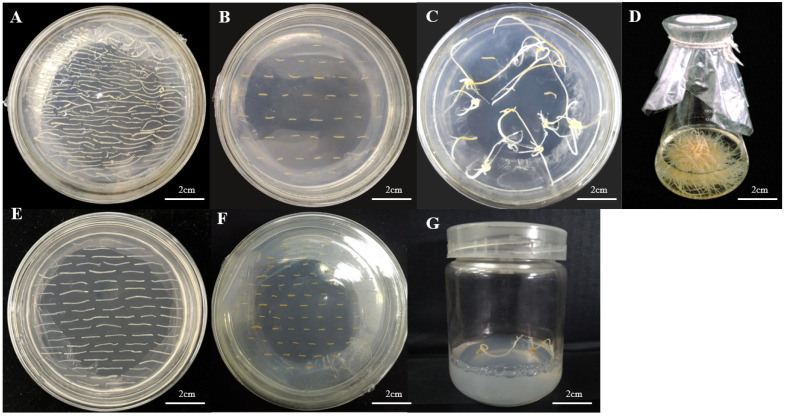
**The process of *PgCYP309* gene overexpression vector transformation and interference vector transformation.** (**A**) Precultured ginseng adventitious root; (**B**) infection of adventitious roots by *Agrobacterium tumefaciens* A4 containing *PgCYP309* gene; (**C**) induced hairy roots; and (**D**) positive hairy roots of propagation. (**E**) Precultured ginseng adventitious root; (**F**) A4-infected adventitious root containing *PgCYP309* interference fragment; and (**G**) induced hairy roots.

Adventitious roots were infested using engineered bacteria containing pBI121-PgCYP309 and pART27-PgCYP309. After the dark culture for a period of time, hairy roots were grown, and after the length of hairy roots reached 1–1.5 cm, succession culture was carried out, and finally the material was expanded by the 250 mL shake flask culture. The process of transforming ginseng adventitious roots is shown in Figure 6. Among the 2000 pieces of transformed material transferred into the *PgCYP309* gene overexpression vector, a total of 22 hairy roots were obtained, with an induction rate of 1.1%. Among the 1800 pieces of transformed material transferred into the *PgCYP309* gene disruption vector, a total of 15 hairy roots were obtained, with an induction rate of 0.8%. Genomic DNA was extracted from a single root system and validated by triple PCR using ddH_2_O as a blank control. A total of five overexpression-positive monocot lines were obtained, with a transformation rate of 22.7%; six interference-positive monocot lines were obtained, with a transcription rate of 40%. Only the saponin and gene expression of the overexpression-positive material was tested because the slow growth and low accumulation of the interference-positive material prevented subsequent experiments.

### 3.7. Detection of Gene Expression and Saponin Content in Positive Hair Roots

**Figure 7:**
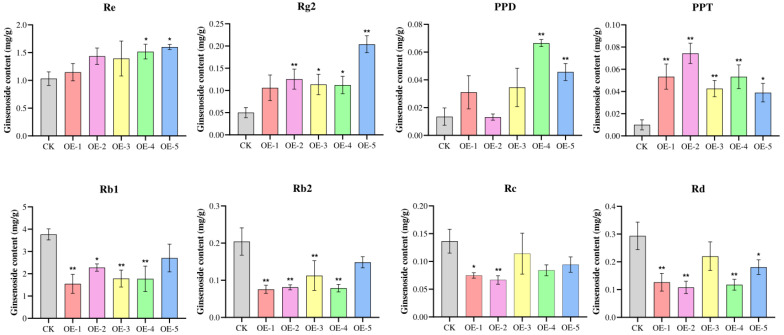
**Ginsenoside content in ginseng hairy roots with overexpression of *PgCYP309* gene.** The X-axis indicates the single root system of ginseng hair root with positive overexpression. The Y-axis indicates the ginsenoside content (mg/g). CK: control check. “*” indicates significant difference when *p* ≤ 0.05. “**” indicates significant difference when *p* ≤ 0.01.

Ginsenosides from overexpressing positive hairy roots were extracted, and the contents of 12 monomeric saponins, including Rb1, Rb2, Rb3, Rc, Rd, Re, Rf, Rg1, Rg2, Rg3, Rh1, and Rh2, as well as two sapogenins, PPD and PPT, were determined using high-performance liquid chromatography (HPLC). The resulting saponin content was calculated and the saponins in which there was a significant change are shown in Figure 7 and Table S2. PPD, PPT, and PPT-type monosaponins Re and Rg2 contents increased significantly, while PPD-type monosaponins Rb1, Rb2, Rc, and Rd contents decreased significantly. It is hypothesized that the *PgCYP309* gene may be involved in the synthesis of PPT-type saponins, which compete with the synthesis of PPD-type saponins.

**Figure 8:**
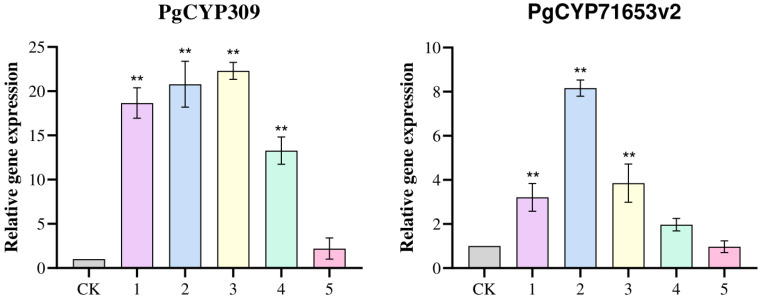
**Expression levels of the *PgCYP309* gene and key enzyme genes in ginsenoside synthesis in positive ginseng hairy roots.** The X-axis indicates the number of ginseng hairy roots. The Y-axis indicates the relative expression of genes. CK: control check. “**” indicates significant at *p* ≤ 0.01.

The relative expression levels of the *PgCYP309* gene and six key enzyme genes (*PgDDS*, *PgCYP716A47*, *PgCYP716A53v2*, *PgCYP716A52v2*, *PgUGT1*, and *PgUGT100*) were detected and analyzed computationally by using qRT-PCR. The results are shown in Figure 8, in which the relative expression of two genes, *PgCYP309* and *PgCYP716A53v2*, was significantly elevated, whereas the other genes did not change significantly. Combined with the analysis of the saponin content results, it was hypothesized that the *PgCYP309* gene may promote the synthesis of PPT-type saponins and have a synergistic effect with the *PgCYP716A53v2* gene.

## 4. Discussion

The preliminary work of this study is based on the fact that the *CYP450* gene family in Jilin ginseng has already been mined by bioinformatics, and the structure, function, evolution, and interactions network of this family of genes have been systematically analyzed. Five candidate genes related to ginsenoside biosynthesis were screened, namely *PgCYP205*, *PgCYP234*, *PgCYP300*, *PgCYP309*, and *PgCYP361*. In order to screen for genes more likely to be involved in ginsenoside biosynthesis from these five genes, in this study, we first analyzed the correlation between the five candidate genes and ginsenoside content and found that all five genes were highly significantly correlated with total saponins. The *PgCYP205* gene was highly significantly correlated with three monomeric saponins; the *PgCYP234* gene was highly significantly correlated with three monomeric saponins; the *PgCYP300* gene was highly significantly correlated with one monomeric saponin; the *PgCYP309* gene was highly significantly correlated with five monomeric saponins; and the *PgCYP361* gene was highly significantly correlated with two monomeric saponins. Then, these five genes were analyzed for interactions with key enzyme genes, and it was found that four genes, *PgCYP205*, *PgCYP300*, *PgCYP309*, and *PgCYP361*, were clustered in the same cluster with the key enzyme genes at *p* value ≤ 5.00 × 10^−2^ and *p* value ≤ 1.00 × 10^−2^, respectively. At *p* value ≤ 1.00 × 10^−3^ and *p* value ≤ 1.00 × 10^−4^, three genes, *PgCYP300*, *PgCYP309*, and *PgCYP361*, were clustered in the same cluster as the key enzyme genes. Combining the results of correlation analysis and the results of reciprocal analysis, the *PgCYP309* gene was associated with the largest variety of monomeric saponins among the five genes. Moreover, the gene was always clustered in the same cluster with the key enzyme genes at progressively stricter *p* values, so finally PgCYP309 was identified as the target gene.

So far, several reports have shown that methyl jasmonate-induced treatment strongly activates the biosynthesis of triterpenoid saponins in many species, including ginseng [27,38]. In this study, methyl jasmonate was used to induce adventitious roots of ginseng, and the induction treatment was started when the adventitious roots were in their best condition at 25 days of growth. The results showed that MeJA induction resulted in a significant increase in PPD-type saponins in ginseng adventitious roots, whereas there was no significant change in PPT-type saponins, which is in general agreement with the study reported by Kim et al. [39]. It has been found that appropriate concentrations of MeJA have a regulatory effect on the transcriptional expression levels of genes involved in plant secondary metabolic processes [40,41]. In this study, we also analyzed the expression levels of the target gene *PgCYP309* and six key enzyme genes after MeJA induction using fluorescence quantification. Based on the results, it was hypothesized that the six genes, *PgCYP309*, *PgDDS*, *PgCYP716A47*, *PgCYP716A53v2*, *PgUGT1* and *PgUGT100*, might begin to exercise their functions in a large number of transcriptional translations at 12 h, and participate in the catalyzing of the synthesis of ginsenosides. At 48 h, the content of ginsenosides started to increase significantly, and only the relative expression of *PgDDS* and *PgUGT1* genes increased significantly, which also proved that the synthesis of ginsenosides is a complex process with the participation of multiple genes in the regulation. The target gene, *PgCYP309*, responded to the induction of MeJA and showed a similar expression activity pattern to that of the key enzyme genes, laying a foundation for subsequent studies.

Currently, 73 *CYP450* genes associated with triterpenoid saponin biosynthesis have been reported [24]. Most of them were functionally analyzed by yeast expression systems, while only three were from ginseng, and these three genes have been validated in both yeast and ginseng for their function of catalyzing ginsenoside synthesis [29,30,42]. Therefore, in this study, we identified one *CYP450* gene (*PgCYP309)*, which is highly related to ginseng saponins, on the basis of our previous work, and functionally validated it in the hope of revealing its role in saponin synthesis. In positive hairy roots overexpressing the *PgCYP309* gene, the contents of both PPD and PPT sapogenins were significantly increased, but the contents of PPD-type monomeric saponins Rb1, Rb2, Rc, and Rd were significantly decreased, whereas the contents of PPT-type monomeric saponins Re and Rg2 were significantly increased. The results of qRT-PCR experiments showed that the relative expression of *PgCYP309* gene was significantly increased in positive hairy roots overexpressing *PgCYP309* gene and the relative expression of the key enzyme gene, *PgCYP716A53v2* has been shown to catalyze the generation of PPT from PPD [29]. The ORF length of the *PgCYP716A53v2* gene was 1410 bp, and the ORF similarity to the *PgCYP309* gene with a length of 1563 bp was 35.38%. So, we envisioned two possibilities, one could be that the *PgCYP309* gene acts as a new gene to catalyze PPD generation of PPT along with the *PgCYP716A53v2* gene. Another possibility is that the *PgCYP309* gene catalyzes the generation of PPD from dammarenodiol, which provides a substrate for catalysis by the *PgCYP716A53v2* gene. But both of these possibilities need to be verified by more and more in-depth research. It can only be determined that the *PgCYP309* gene promotes dammarane-type saponin synthesis. In addition, it is unfortunate that due to the low accumulation of interference-positive material in this study, the examination of saponin content and relative gene expression was not possible for the time being.

## 5. Conclusions

In this study, through the systematic analysis of the correlation between the five *PgCYP450* genes obtained in the pre-laboratory stage and ginsenoside content, as well as the interactions with the genes of key enzymes for ginsenoside synthesis. *PgCYP309* gene was screened as the target gene and analyzed for its physicochemical properties. Methyl jasmonate-induced treatment of ginseng adventitious roots revealed that the *PgCYP309* gene was involved in ginsenoside synthesis in response to methyl jasmonate induction. The *PgCYP309* gene was successfully cloned, and the overexpression vector and interference vector were constructed. The ginseng adventitious roots were transformed by the *Agrobacterium fermentum*-mediated method to obtain positive ginseng hairy roots. The conversion rate of hairy roots overexpressing the *PgCYP309* gene was 22.7% and the conversion rate of hairy roots interfering with the *PgCYP309* gene was 40%. The *PgCYP309* gene was found to be involved in the synthesis of ginsenosides by the detection of positive hairy root saponin content and relative gene expression, and it was preliminarily verified that the *PgCYP309* gene promotes the synthesis of dammarane-type ginsenosides.

## Data Availability

All data generated or analyzed during this study are included in this published article. All plant materials are available through corresponding authors upon request.

## Supplementary Materials

The following supporting information can be downloaded at: https://www.mdpi.com/article/10.3390/biom14060715/s1, Table S1: Ginsenoside content data of samples treated with methyl jasmonate; Table S2: Ginsenoside content data of *PgCYP309* gene overexpression positive materials of ginseng hairy roots.

## Abbreviations

PPD	protopanaxadiol
PPT	protopanaxatriol
MeJA	Methyl jasmonate
RT-qPCR	Quantitative real-time PCR
ddH_2_O	Double distilled water
nm	Nanometer
TPS	Extraction solution (Tris-HCl, EDTA, KCl, ddH_2_O)
B5	Gamborg B5
MS	Murashige and skoog
OD	Optical density
AS	Acetosyringone
